# Total cholesterol and high density lipoprotein cholesterol ratio is associated with metabolic syndrome in a very elderly Chinese population

**DOI:** 10.1038/s41598-022-19445-5

**Published:** 2022-09-08

**Authors:** Gang Huang, Junbo Xu, Zhen Zhang, Lin Cai, Hanxiong Liu, Xiuqiong Yu

**Affiliations:** 1 Department of Cardiology, The Third People’s Hospital of Chengdu, No. 82, Qinglong Street, Qingyang District, Chengdu, 610031 Sichuan China; 2 Cardiovascular Disease Research Institute of Chengdu, Chengdu, Sichuan China; 3 Affiliated Hospital of Southwest Jiaotong University, Chengdu, Sichuan China; 4 The Second Affiliated Chengdu Clinical College of Chongqing Medical University, Chengdu, Sichuan China

**Keywords:** Risk factors, Metabolic disorders, Geriatrics, Dyslipidaemias

## Abstract

Metabolic syndrome (MetS) is currently a major public health challenge in young, middle aged and elderly population worldwide, but it is still not clear in very elderly population. This study was to investigate the potential association between total cholesterol and high density lipoprotein cholesterol ratio (THR) and MetS in a very elderly population in Chengdu. Totally, 1056 very elderly (aged ≥ 80 years) in Chengdu community were enrolled in this study. Geographic characteristics of participants were collected and laboratory measurement was performed. Metabolic syndrome (MetS) was defined according to the Chinese and the international diabetes federation (IDF) criteria, respectively. Logistic analysis was used to investigate the potential association between the THR and MetS. Receiver operating characteristic curve (ROC) analysis was used to evaluate the efficiency of THR in MetS predicting. Finally, 1038 participants were included in statistical analysis. The mean age was 83.6 ± 3.4 years and 52.6% participants were men and 21.6% suffered from MetS. Participants with MetS had relatively higher waist circumference, body weight, blood pressure, fast plasma glucose level, non-high density lipoprotein cholesterol level and THR. The logistic analysis revealed that the THR was associated with MetS according to both the Chinese (odds ratio (OR): 3.053, 95% confidence interval (CI) 2.464–3.782, P < 0.001) and the IDF criteria (OR: 2.458, 95% CI 2.016–2.995, P < 0.001). ROC analysis found that the area under curve of the THR was 0.800 (95% CI 0.749–0.852, P < 0.001) and 0.727 (95% CI 0.669–0.786, P < 0.001) for predicting MetS according to the Chinese and the IDF criteria, respectively. The THR is associated with MetS in this community very elderly population in Chengdu.

## Introduction

Metabolic syndrome (MetS) is nowadays a big challenge worldwide, which is characterized by a cluster of several metabolic disorders, i.e. abdominal obesity, atherogenic dyslipidemia, hypertension and dysregulation of glucose^1,2^. Despite unclear common pathophysiological mechanism, MetS has been recognized to increase the risk of diabetes mellitus (DM) and series atherosclerotic cardiovascular disease(ASCVD), i.e. coronary heart disease^3,4^, cardiovascular mortality^2,5^, cognitive impairment^6^ and all cause mortality^7^. Lifestyle modification and risk factors management are currently recommended to decrease the risk of subsequent cardiovascular diseases. Previous studies ^8,9^ have emphasized the importance of dyslipidemia as one component for the diagnosis of MetS and a recent epidemiological study^10^ has demonstrated that more than one third the Chinese adults younger than 75 years in 2010 suffered from MetS, which was similar to the epidemiological situation in the USA in 2014. Furthermore, the prevalence of MetS in the Americans older than 60 years was 1.6-fold higher^11^, which has shown that MetS components are more likely to cluster together in the older Americans. Dyslipidemia (hypertriglyceridemia and hypo-high density lipoproteinemia) is an important component for MetS, which plays a critical role in the progression from MetS to DM and ASCVD ^8,9^. Several studies^12–18^ have investigated the potential ability of the total cholesterol to high-density lipoprotein cholesterol (THR) and triglyceride to high-density lipoprotein cholesterol ratio to predict ASCVD, stroke and diabetes in adolescents, young and middle aged population, while few^19,20^ study has reported this potential association in very elderly until now.

Therefore, this study aimed to investigate the potential association between the THR and MetS in a community very elderly population.

## Methods

### Study population

This study was designed to investigate cardiovascular and metabolic risk factors in general community very elderly (≥ 80 years old) in Chengdu, which locates in the southwest of China^21^. From 2013 to 2015, a representative sample of very elderly in communities were recruited by using of a stratified three-stage cluster sampling design, which was described previously elsewhere^21^. Totally, 1056 very elderly from 20 residential communities were enrolled according to registration data from the local government. The study protocol conforms to the ethical guidelines of the 1975 Declaration of Helsinki as reflected in a prior approval by the ethics committee of the second people’s hospital of Chengdu. And all participants have given informed consent.

### Demographic data collection and laboratory test

Well trained physicians and nurses were responsible for demographic data collecting (e.g. medical history, lifestyle, cardiovascular and metabolic risk factors) by a questionnaire-based face to face interview with a standardized questionnaire. The body mass index (BMI) was defined as weight in kilograms divided by the square of the height in meters. Blood pressure (BP) were measured three times in a sitting position by using a standardized automatic electronic sphygmomanometer (HEM-7300, Omron, Kyoto, Japan) according to the Chinese Guidelines for the Management of Hypertension^22^ and average values were calculated and included in the statistical analysis.

After fasting at least for 8 h, blood samples were collected from all participants and biochemical parameters, e.g. fast plasma glucose (FPG), total cholesterol (TC), triglycerides (TG), low-density lipoprotein cholesterol (LDL-C), high-density lipoprotein cholesterol (HDL-C), creatinine and serum uric acid were analyzed enzymatically on an auto-analyzer (AU5421 Chemistry Analyzer, Beckman, Brea, California, United States) in the central laboratory of our hospital.

The estimated glomerular filtration rate (eGFR) was calculated by using the Modification of Diet in Renal Disease study equation modified for the Chinese: eGFR = 186 × serum creatinine^−1.154^ × Age^−0.203^ × 0.742 (if women).

### Diagnostic criteria of MetS

In this study, MetS was defined according to the Chinese guideline for dyslipidemia management^23^ and the Consensus Worldwide Definition from International Diabetes Federation (IDF)^24^ respectively as follows.

Chinese criteria: MetS should fulfill any three or more of the following items: abdominal obesity (waist circumference (WC) ≥ 90 cm in men and ≥ 85 cm in women), fasting TG ≥ 150 mg/dL (1.7 mmol/L), fasting HDL-C < 40 mg/dL (1.0 mmol/L), FPG ≥ 110 mg/dL (6.10 mmol/L) or 2 h blood glucose after glycemic load ≥ 140 mg/dL (7.80 mmol/L) or anti-diabetic treatment, and BP ≥ 130/85 mmHg or anti-hypertensive treatment.

IDF criteria: abdominal obesity with ethnic-specific WC cut-points (≥ 90 cm for Chinese men and ≥ 80 cm for women) and fulfills two items of the following: TG ≥ 150 mg/dL (1.7 mmol/L) or treatment for hypertriglycerides, HDL-C < 40 mg/dL (1.03 mmol/L) in men or < 50 mg/dL (1.29 mmol/L) in women or treatment for low HDL-C, FPG ≥ 100 mg/dL (5.6 mmol/L) or previously diagnosed type 2 diabetes, and BP ≥ 130/85 mmHg or treatment for hypertension.

### Statistical analysis

All statistical analysis were performed by using SPSS software (Version 22.0, SPSS Inc, Chicago, IL). Continuous variables are expressed as mean ± standard deviation and frequencies are presented as percentages. Statistical comparison of continuous variables between groups was conducted using ANOVA or Kruskal–Wallis test, whereas χ^2^ test was applied to compare frequencies. Logistic regression models were used to evaluate the potential association between the THR and MetS. The receiver operating characteristic curve (ROC) analysis was used to evaluate the efficiency of the THR in predicting MetS according to different criteria. A two-sided P value < 0.05 was considered statistically significant.

### Ethics statement

The study protocol conforms to the ethical guidelines of the 1975 Declaration of Helsinki as reflected in a prior approval by the ethics committee of the second people’s hospital of Chengdu and all participants provided written informed consent to participate in this study.

## Results

### Baseline characteristics

Totally, there were 1056 participants enrolled in this study and 1038 of them were included in the final statistical analysis. In this study population, more very elderly women suffered from MetS (Women vs. Men: 33.6% vs. 17.3% (IDF criteria), 25.8% vs. 17.8% (Chinese criteria)). The mean THR was 3.25 ± 0.93 (men vs. Women: 3.27 ± 0.93 vs. 3.24 ± 0.92, P = 0.508). Moreover, participants with MetS were relatively younger and more likely to smoke and drink currently. And they had relatively higher WC, body weight, BP, FPG, non-HDL-C, serum uric acid, THR and lower eGFR levels (Table 1).

**Table 1:** Baseline characteristics of very elderly according to MetS (Chinese criteria).

	MetS (n = 224)	No MetS (n = 814)	P value
Age (years)	83.06 ± 2.90	83.71 ± 3.49	0.044
Male, n (%)	97 (43.3)	449 (55.2)	0.002
Current smoking, n (%)	27 (12.1)	88 (10.8)	0.649
Current drinking, n (%)	21 (9.4)	65 (8.0)	0.565
**Medical history, n (%)**			
Hypertension	138 (61.6)	408 (50.1)	0.005
DM	71 (31.7)	107 (13.1)	< 0.001
Abdominal obesity, n (%)	202 (90.2)	272 (33.4)	< 0.001
**Medication, n (%)**			
Antihypertensive	126 (56.2)	344 (42.3)	0.834
Antidiabetic	62 (27.6)	77 (9.4)	0.027
Lipid lowering	25 (11.2)	61 (7.5)	0.615
WC (cm)	95.10 ± 7.29	85.09 ± 10.30	< 0.001
Height (cm)	154.78 ± 10.11	155.01 ± 10.11	0.512
Body weight (kg)	60.81 ± 9.80	54.05 ± 10.74	< 0.001
BMI	25.34 ± 3.35	22.42 ± 3.57	< 0.001
SBP (mmHg)	152.9 ± 18.8	146.1 ± 22.9	< 0.001
DBP (mmHg)	75.8 ± 10.6	74.1 ± 12.4	0.030
FPG (mmol/L)	7.21 ± 2.85	5.28 ± 1.38	< 0.001
TC (mmol/L)	5.05 ± 1.02	4.84 ± 0.99	0.008
TG (mmol/L)	2.10 ± 1.09	1.19 ± 0.61	< 0.001
LDL-C (mmol/L)	2.81 ± 0.74	2.53 ± 0.74	< 0.001
HDL-C (mmol/L)	1.33 ± 0.36	1.67 ± 0.43	< 0.001
THR	4.00 ± 0.96	3.03 ± 0.79	< 0.001
SUA (µmol/L)	375.21 ± 88.84	350.66 ± 96.01	< 0.001
Creatinine (μmol/L)	109.05 ± 41.40	102.81 ± 29.72	0.231
eGFR (mL/(min∙1.73 m^2^))	55.14 ± 15.08	56.40 ± 18.41	0.044

Data are expressed as mean ± standard deviation for continuous variables or number (percentage) for categorical variables.

*BMI* body mass index, *DBP* diastolic blood pressure, *DM* diabetes mellitus, *eGFR* estimated glomerular filtration rate, *FPG* fast plasma glucose, *HDL* high-density lipoprotein cholesterol, *LDL* low-density lipoprotein cholesterol, *SUA* serum uric acid, *SBP* systolic blood pressure, *TC* total cholesterol, *TG* triglyceride, *THR* total and high density lipoprotein cholesterol ratio, *WC* Waist circumference.

Participants with lower tertile category of THR had a lower estimated prevalence of MetS and its components (i.e. abdominal obesity, hypertirglyceridemia, low HDL cholesterol and hyperglycemia) according both to the Chinese and the IDF criteria (Table 2).

**Table 2:** Estimated prevalence of metabolic syndrome and its components.

	Tertile categories of THR							
	Chinese criteria				IDF criteria			
	T1 (n = 346)	T2 (n = 346)	T3 (n = 346)	P for trends	T1 (n = 346)	T2 (n = 346)	T3 (n = 346)	P for trends
MetS, n (%)	22 (6.4)	47 (13.6)	155 (44.8)	< 0.001	40 (11.6)	63 (18.2)	157 (45.4)	< 0.001
Abdominal Obesity, n (%)	148 (42.8)	170 (49.1)	232 (67.1)	< 0.001	176 (50.9)	196 (56.6)	246 (71.1)	< 0.001
High blood pressure, n (%)	271 (78.3)	266 (76.9)	293 (84.7)	0.024	271 (78.3)	266 (76.9)	293 (84.7)	0.024
Hypertirglyceridemia,n (%)	15 (4.3)	45 (13.0)	173 (50.0)	< 0.001	15 (4.3)	45 (13.0)	173 (50.0)	< 0.001
Low HDL cholesterol, n (%)	2 (0.6)	4 (1.2)	65 (18.8)	< 0.001	6 (1.7)	19 (5.5)	129 (37.3)	< 0.001
Hyperglycemia, n (%)	54 (15.6)	75 (21.7)	115 (33.2)	< 0.001	96 (27.7)	103 (29.8)	146 (42.2)	< 0.001

Data are expressed as number (percentage).

*HDL* high-density lipoprotein, *IDF* international diabetes federation, *MetS* metabolic syndrome, *THR* total and high density lipoprotein cholesterol ratio. The cut-off values of THR are 2.72 and 3.50.

### Logistic regression analysis of the MetS risk

**Figure 1:**
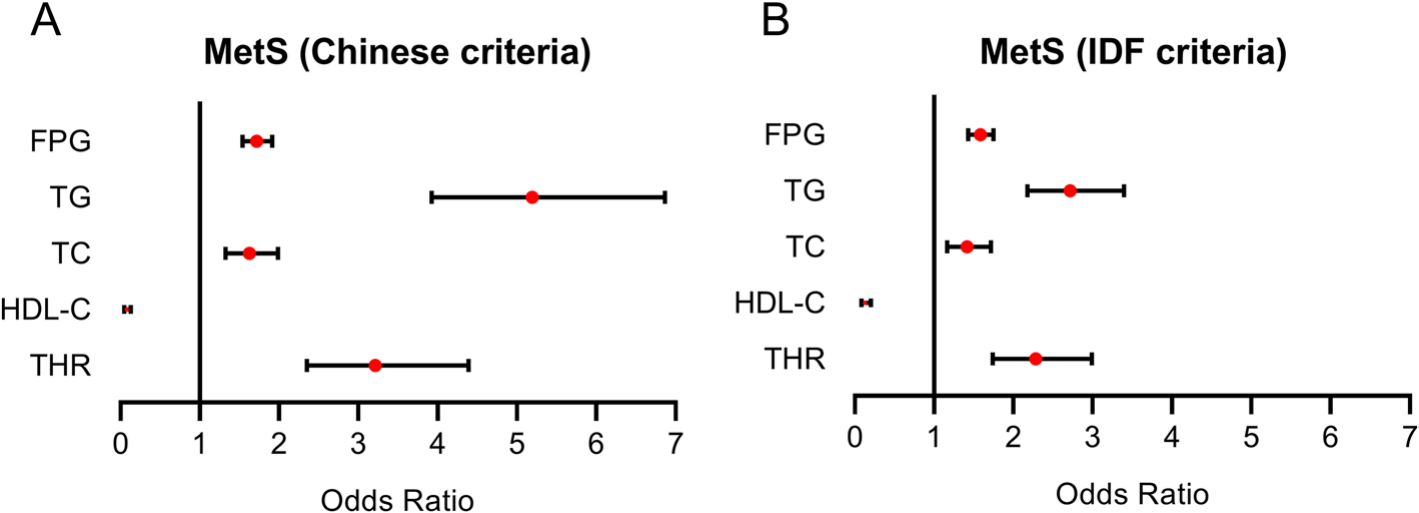
Logistic regression results of laboratory parameters for MetS predicting. (**A**) MetS according to the Chinese criteria. (**B**) MetS according to the IDF criteria. *FPG* fast plasma glucose, *HDL-C* high-density lipoprotein cholesterol, *IDF* international diabetes federation, *MetS* metabolic syndrome, *TC* total cholesterol, *TG* triglyceride, *THR* total cholesterol and high density lipoprotein cholesterol ratio.

The logistic regression analysis found that FPG, TC, TG, and HDL-C were associated with MetS (Fig. 1). The THR was associated with an increased risk of MetS according both to the Chinese criteria (odds ratio (OR): 3.211, 95% confidence interval (CI) 2.349–4.388, P < 0.001) and the IDF criteria (OR: 2.281, 95% CI 1.742–2.989, P < 0.001) in this very elderly population. After adjustment of sex, BMI, hyperurecima and eGFR, the THR was found to be also associated with an increased risk of MetS according both to the Chinese criteria (OR: 3.107, 95% CI 2.507–3.849, P < 0.001) and the IDF criteria (OR: 2.418, 95% CI 1.981–2.951, P < 0.001). Furthermore, the THR was found to be still associated with the increase of MetS according both to the Chinese criteria (OR: 3.053, 95% CI 2.464–3.782, P < 0.001) and the IDF criteria (OR: 2.458, 95% CI 2.016–2.995, P < 0.001) in this very elderly population after adjustment of sex, age, BMI, hyperurecimia and eGFR, current smoking, current drinking and physical activity (Table 3).

**Table 3:** Association between the THR and MetS according to models with different risk factors.

	MetS (Chinese criteria)		MetS (IDF criteria)	
	OR (95% CI)	P value	OR (95% CI)	P value
Model 1	3.211 (2.349–4.388)	< 0.001	2.281 (1.742–2.989)	< 0.001
Model 2	3.107 (2.507–3.849)	< 0.001	2.418 (1.981–2.951)	< 0.001
Model 3	3.053 (2.464–3.782)	< 0.001	2.458 (2.016–2.995)	< 0.001

Model 1: not adjusted.

Model 2: adjusted for sex. BMI, hyperurecemia and eGFR.

Model 3: adjusted for sex, age, BMI, hyperurecemia and eGFR, current smoking, current drinking and physical activity.

*BMI* body mass index, *CI* conference interval, *eGFR* estimated glomerular filtration rate, *IDF* international diabetes federation, *MetS* metabolic syndrome, *OR* odds ratio, *THR* total and high density lipoprotein cholesterol ratio.

### ROC analysis of MetS predicting

**Figure 2:**
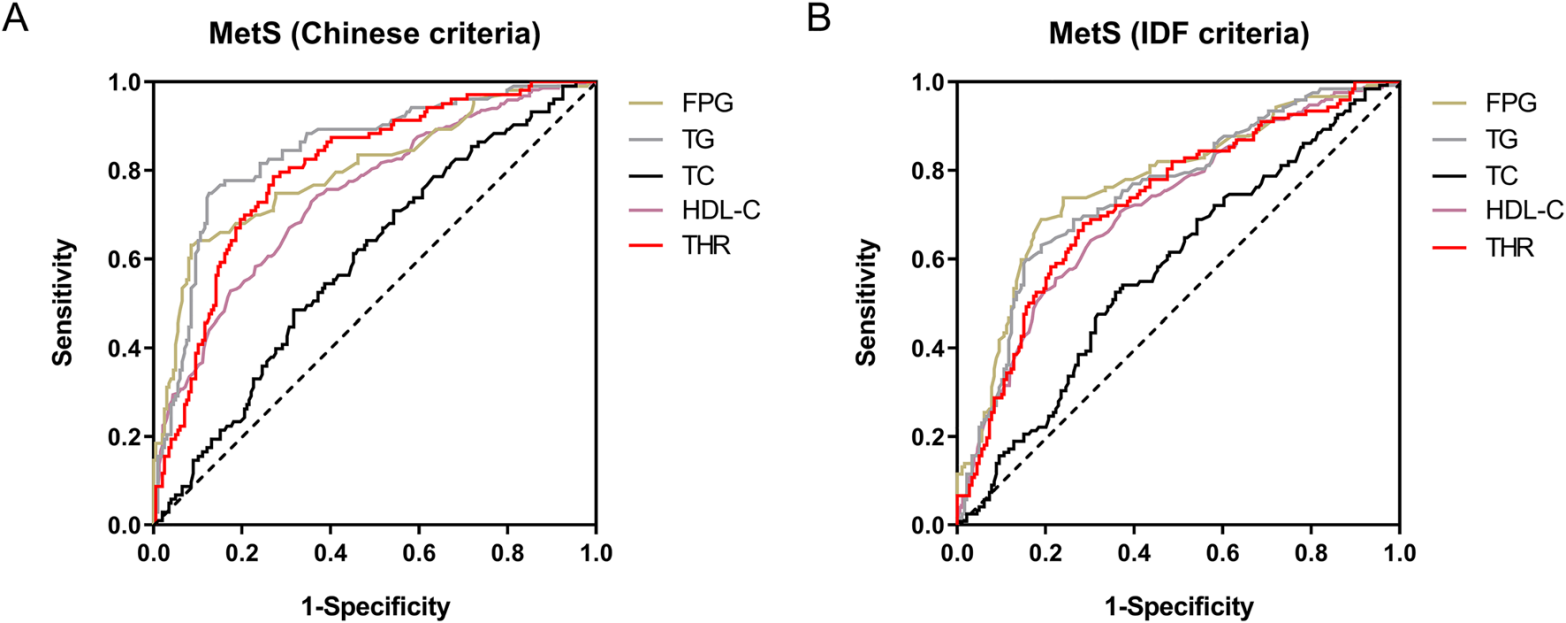
ROC curves of different parameters for MetS predicting. (**A**) ROC analysis of the THR for MetS predicting according to the Chinese criteria. The AUC of THR was 0.800 (95% CI 0.749–0.852). (**B**) ROC analysis of the THR for MetS predicting according to the IDF criteria. The AUC of the THR was 0.727 (95% CI 0.669–0.786). *AUC* area under the ROC curve, *FPG* fast plasma glucose, *HDL-C* high-density lipoprotein cholesterol, *IDF* international diabetes federation, *MetS* metabolic syndrome, *ROC* Receiver operating characteristic, *TC* total cholesterol, *TG* triglyceride, *THR* total cholesterol and high density lipoprotein cholesterol ratio.

ROC analysis found that the area under the ROC curve (AUC) of the THR was 0.800 (95% CI 0.749–0.852, P < 0.001) and 0.727 (95% CI 0.669–0.786, P < 0.001) for predicting MetS in very elderly according to the Chinese and the IDF criteria, respectively. Moreover, the AUC of THR for predicting MetS (Chinese criteria) was similar to that of FPG (0.800, 95% CI 0.745–0.856, P < 0.001), lower than TG (0.843, 95% CI 0.795–0.8961, P < 0.001) and higher than HDL-C (0.744, 95% CI 0.706–0.781, P < 0.001). The AUC of TG was the biggest for predicting MetS according to the Chinese criteria (0.843, 95% CI 0.795–0.891, P < 0.001), while the AUC of FPG was the biggest according to the IDF criteria (0.771, 95% CI 0.715–0.826, P < 0.001) (Fig. 2).

## Discussion

### Dyslipidemia and MetS

It is well known that MetS is a cluster or combination of several metabolic abnormalities without fully understood pathogenesis currently^25^. Framingham Heart Study (FHS)^26^ has demonstrated that the prevalence of MetS is two to three times higher in Framingham residents aged 50 years and older than in younger ages. Unclassified interaction between genetic and environmental factors may play an important role in the pathological process of MetS. Genetic variants in MetS are associated especially with glucose metabolism or lipid metabolism. And genetic susceptibility may exist within adipose tissue, in insulin signaling pathways, and in regulation of individual components of MetS. Insulin resistance or hyperinsulinaemia may contribute to obesity- and DM related hypertension and possibly also promote dyslipidaemia in MetS. Obesity, lifestyle, chronic inflammation and circadian rhythm disturbances may also contribute to the genesis of MetS^27,28^. Dyslipidaemia in MetS is mainly characterized by highly atherogenic small dense low-density lipoprotein and small dense high-density lipoprotein particles which can deplete of triglyceride^9^. One recent study^29^ has identified more than thirty new lipids contributing to key metabolic risk factors, i.e. obesity, dyslipidemia and dysglycemia in FHS participants. Triglyceride could result in endothelial dysfunction and foam cells formation by accelerating the oxidation of LDL-C. While in contrast, HDL-C play an anti-atherosclerosis role through transporting excess cholesterol to liver. Therefore, hypertriglyceridemia and hypo-high density lipoproteinemia play an important role in the pathogenesis in MetS. A previous study^30^ has reported that hypertriglyceridemia with a prevalence of 10.8% is the main type of lipid disorders in Chinese older than 60 years, especially in older women, which is in line with results of our previous study ^21^. In the present study, all levels of LDL-C, TC and TG, WC and BMI were higher in the very elderly participants than in Chinese adults aged 18–74 years, while FPG level was similar to that of general adults^10,21^. Potential reasons include typical local dietary style rich in oil and physical inactivity in population in this area. Especially, the prevalence of abdominal obesity and hypertriglyceridemia was even higher in this very elderly population than that of Chinese adults aged 18–74 years and middle aged population in this area. Moreover, the prevalence of hypercholesteremia is notably higher than that of hypertriglyceridemia^21^. Aside from high prevalence of hypertension, these dramatic characteristics and clustering of components mentioned above contribute significantly to the relatively high prevalence of MetS in this very elderly population^31^. However, different criteria of MetS chosen among studies, different constitution of study population and survey duration may contribute to the prevalence difference of MetS among studies.

### THR and MetS

It has been reported that the THR is associated with an increased risk of ischemic stroke of middle aged to elderly community residents in FHS^19^ and in Chinese men aged 20–80 years^18^. Also, a study^20^ including nearly 3000 participants older than 60 years has found that the THR is associated with low ankle brachial index in non-smokers and therefore THR maybe play a role in peripheral arterial disease early screening. In Sweden women aged 50–59 years, the THR has been found to have a predictive ability for ischemic heart disease as well^13^. Vischer et al.^32^ has found that low TC and HDL-C levels can predict total mortality in very elderly French. Among very elderly participants in this study, TC level and the THR were significantly higher and HDL-C level was significantly lower in participants with MetS. Previous studies ^12,13,33,34^ have already demonstrated that the THR and the TG/HDL-C ratio are associated with MetS in general younger population. This current study has also investigated that there is an association between the THR and MetS according both to the Chinese and the IDF criteria in very elderly population. However, statistical analyses have demonstrated that THR has a higher ability for MetS prediction according to the Chinese criteria than the IDF criteria. And interestingly, very elderly women are more likely to suffer from MetS according to both criteria in this very elderly population, which is different from the results of a previous study in young and middle aged Chinese^30^. Although the Chinese MetS criteria are mainly different from the IDF criteria in WC and HDL cholesterol cut-off points, the prevalence of abdominal obesity, hypercholesterolaemia and hypertriglyceridemia in this very elderly women population are higher than that of young and middle aged women, which may be one of the potential explanations for the difference of MetS predicting according to different MetS criteria.

It’s not surprising that this study has also demonstrated that the ability of the THR for predicting of MetS is similar to that of FPG and it is somewhat inferior to which of TG in this very elderly population. TG and FPG abnormalities are important components of MetS according to different criteria. Moreover, they could be somewhat more easily influenced by diets before testing. Therefore, except for these direct measured parameters for MetS diagnosis, the THR could be a reliable indirect measured parameter for MetS predicting in very elderly before absolute changes of directly measured lipid parameters become apparent or could be consider as an potential alternative marker for MetS in daily clinical practice.

### Study limitations

Several limitations should be considered in this study. First, this cross-sectional study could not describe any causality. Second, the study population comes from the southwest of China, whether current findings could be generalized to other very elderly population or younger population in other areas of China needs further clarification from further longitudinal prospective studies, although limited life expectancy of very elderly could restrict the conduction of perspective studies in this special population.

## Conclusions

In conclusion, the main finding of this study is that THR is associated with the increase risk of MetS and it could potentially add diagnostic information for MetS in very elderly Chinese population. Based on the high prevalence of abdominal obesity, dyslipidemia and glucose abnormality in this study and the oil rich local daily food style, proper lifestyle modification, especially a dietary modification are still needed to be emphasized in the prevention of MetS and ASCVD in very elderly, although the residual life expectancy of very elderly population is not the same long as that of younger population.

## Abbreviations

ASCVD: Atherosclerotic cardiovascular disease
AUC: Area under the ROC curve
CI: Confidence interval
DM: Diabetes mellitus
eGFR: Estimated glomerular filtration rate
FPG: Fast plasma glucose
FHS: Framingham Heart Study
HDL-C: High-density lipoprotein cholesterol
IDF: International Diabetes Federation
LDL-C: Low-density lipoprotein cholesterol
MetS: Metabolic syndrome
OR: Odds ratio
ROC: Receiver operating characteristic curve
THR: Total and high density lipoprotein cholesterol ratio
TC: Total cholesterol
TG: Triglycerides
WC: Waist circumference

## References

[1] Eckel RH, Grundy SM, Zimmet PZ (2005). The metabolic syndrome. Lancet.

[2] Saklayen MG (2018). The global epidemic of the metabolic syndrome. Curr. Hypertens. Rep..

[3] Dugani SB, Moorthy MV, Li C (2021). Association of lipid, inflammatory, and metabolic biomarkers with age at onset for incident coronary heart disease in women. JAMA Cardiol..

[4] Lind L, Sundström J, Ärnlöv J, Risérus U, Lampa E (2021). A longitudinal study over 40 years to study the metabolic syndrome as a risk factor for cardiovascular diseases. Sci. Rep..

[5] Lakka HM, Laaksonen DE, Lakka TA (2002). The metabolic syndrome and total and cardiovascular disease mortality in middle-aged men. JAMA.

[6] Pal K, Mukadam N, Petersen I, Cooper C (2018). Mild cognitive impairment and progression to dementia in people with diabetes, prediabetes and metabolic syndrome: A systematic review and meta-analysis. Soc. Psychiatry Psychiatr. Epidemiol..

[7] Ford ES (2005). Risks for all-cause mortality, cardiovascular disease, and diabetes associated with the metabolic syndrome: A summary of the evidence. Diabetes Care.

[8] Barkas F, Elisaf M, Liberopoulos E, Liontos A, Rizos EC (2016). High triglyceride levels alter the correlation of apolipoprotein B with low- and non-high-density lipoprotein cholesterol mostly in individuals with diabetes or metabolic syndrome. Atherosclerosis.

[9] Adiels M, Olofsson SO, Taskinen MR, Borén J (2008). Overproduction of very low-density lipoproteins is the hallmark of the dyslipidemia in the metabolic syndrome. Arterioscler. Thromb. Vasc. Biol..

[10] Lu J, Wang L, Li M (2017). Metabolic syndrome among adults in China: The 2010 China Noncommunicable Disease Surveillance. J. Clin. Endocrinol. Metab..

[11] Shin D, Kongpakpaisarn K, Bohra C (2018). Trends in the prevalence of metabolic syndrome and its components in the United States 2007–2014. Int. J. Cardiol..

[12] Gao M, Zheng Y, Zhang W, Cheng Y, Wang L, Qin L (2017). Non-high-density lipoprotein cholesterol predicts nonfatal recurrent myocardial infarction in patients with ST segment elevation myocardial infarction. Lipids Health Dis..

[13] Calling S, Johansson SE, Wolff M, Sundquist J, Sundquist K (2021). Total cholesterol/HDL-C ratio versus non-HDL-C as predictors for ischemic heart disease: A 17-year follow-up study of women in southern Sweden. BMC Cardiovasc. Disord..

[14] Fernández-Macías JC, Ochoa-Martínez AC, Varela-Silva JA, Pérez-Maldonado IN (2019). Atherogenic index of plasma: Novel predictive biomarker for cardiovascular illnesses. Arch. Med. Res..

[15] Kim J, Shin SJ, Kim YS, Kang HT (2021). Positive association between the ratio of triglycerides to high-density lipoprotein cholesterol and diabetes incidence in Korean adults. Cardiovasc. Diabetol..

[16] Zhou L, Mai J, Li Y, Guo M, Wu Y, Gao X, Wu Y, Liu X, Zhao L (2020). Triglyceride to high-density lipoprotein cholesterol ratio and risk of atherosclerotic cardiovascular disease in a Chinese population. Nutr. Metab. Cardiovasc. Dis..

[17] Chu SY, Jung JH, Park MJ, Kim SH (2019). Risk assessment of metabolic syndrome in adolescents using the triglyceride/high-density lipoprotein cholesterol ratio and the total cholesterol/high-density lipoprotein cholesterol ratio. Ann. Pediatr. Endocrinol. Metab..

[18] Liu X, Yan L, Xue F (2019). The associations of lipids and lipid ratios with stroke: A prospective cohort study. J. Clin. Hypertens. (Greenwich)..

[19] Pikula A, Beiser AS, Wang J, Himali JJ, Kelly-Hayes M, Kase CS (2015). Lipid and lipoprotein measurements and the risk of ischemic vascular events: Framingham Study. Neurology.

[20] Zhan Y, Yu J, Ding R, Sun Y, Hu D (2014). Triglyceride to high density lipoprotein cholesterol ratio, total cholesterol to high density lipoprotein cholesterol ratio and low ankle brachial index in an elderly population. Vasa.

[21] Huang G, Xu JB, Zhang TJ (2017). Hyperuricemia is associated with cardiovascular diseases clustering among very elderly women—A community based study in Chengdu, China. Sci. Rep..

[22] Liu LS, Writing Group of 2010 Chinese Guidelines for the Management of Hypertension (2011). 2010 Chinese guidelines for the management of hypertension [in Chinese]. Zhonghua Xin Xue Guan Bing Za Zhi.

[23] Joint Committee for Guideline Revision (2018). 2016 Chinese guidelines for the management of dyslipidemia in adults. J. Geriatr. Cardiol..

[24] International Diabetes Federation. The IDF consensus worldwide definition of the metabolic syndrome. https://www.idf.org/e-library/consensus-statements/60-idfconsensus-worldwide-definitionof-the-metabolic-syndrome.html.(2006) (Accessed 28 January 2022).

[25] Nilsson PM, Tuomilehto J, Rydén L (2019). The metabolic syndrome—What is it and how should it be managed. Eur. J. Prev. Cardiol..

[26] Kraja AT, Borecki IB, North K (2006). Longitudinal and age trends of metabolic syndrome and its risk factors: The Family Heart Study. Nutr. Metab. (Lond.).

[27] Raal FJ (2009). Pathogenesis and management of the dyslipidemia of the metabolic syndrome. Metab. Syndr. Relat. Disord..

[28] Khosravipour M, Khanlari P, Khazaie S, Khosravipour H, Khazaie H (2021). A systematic review and meta-analysis of the association between shift work and metabolic syndrome: The roles of sleep, gender, and type of shift work. Sleep Med. Rev..

[29] Yin X, Willinger CM, Keefe J (2019). Lipidomic profiling identifies signatures of metabolic risk. EBioMedicine.

[30] Wang ZH, Wang LH, Li YC, Zhang M, Hu N, Wang LM (2012). Current status of diabetes, hypertension and dyslipidemia among older Chinese adults in 2010 [in Chinese]. Zhonghua Yu Fang Yi Xue Za Zhi.

[31] Huang G, Xu J, Zhang T (2020). Hyperuricemia is associated with metabolic syndrome in the community very elderly in Chengdu. Sci. Rep..

[32] Vischer UM, Safar ME, Safar H (2009). Cardiometabolic determinants of mortality in a geriatric population: Is there a "reverse metabolic syndrome"?. Diabetes Metab..

[33] Zhang X, Zhang X, Li X, Feng J, Chen X (2019). Association of metabolic syndrome with atherogenic index of plasma in an urban Chinese population A 15-year prospective study. Nutr. Metab. Cardiovasc. Dis..

[34] Zhu Li, Zhan Lu, Zhu L (2015). Lipoprotein ratios are better than conventional lipid parameters in predicting coronary heart disease in Chinese Han people. Kardiol. Pol..

